# Molecular Research on Emerging Viruses: Evolution, Diagnostics, Pathogenesis, and Therapeutics

**DOI:** 10.3390/ijms19020398

**Published:** 2018-01-30

**Authors:** Susanna K. P. Lau

**Affiliations:** 1 State Key Laboratory of Emerging Infectious Diseases, Hong Kong, China; skplau@hku.hk; Tel.: +852-22554892; Fax: +852-28551241; 2 Department of Microbiology, Li Ka Shing Faculty of Medicine, The University of Hong Kong, Hong Kong, China; 3 Carol Yu Centre for Infection, Li Ka Shing Faculty of Medicine, The University of Hong Kong, Hong Kong, China; 4 Collaborative Innovation Center for Diagnosis and Treatment of Infectious Diseases, Li Ka Shing Faculty of Medicine, The University of Hong Kong, Hong Kong, China

## Editorial

Viruses are increasingly recognized as emerging infectious disease agents in both humans and animals. Zoonotic viruses, in particular, often evolve rapidly to adapt to new hosts with enhanced virulence and emerge or re-emerge to cause epidemics. Factors such as urbanization, global warming, and dense human and animal populations may have contributed to the emergence of some viruses. The recent epidemics caused by Zika virus and Middle East respiratory syndrome coronavirus (MERS-CoV) clearly illustrate the ability of emerging viruses to pose huge public health problems within a short time. Much more research effort is needed to understand the evolution and pathogenesis of these emerging viruses, as well as the development of diagnostics and therapeutics to combat existing and future epidemics. 

Recent advances in molecular techniques, such as sequencing and metagenomics, have accelerated our understanding of genetic and host diversity of emerging viruses, and hence their evolutionary pathways. Nevertheless, many questions remain unanswered. For example, while severe acute respiratory syndrome coronavirus (SARS-CoV) was originated from horseshoe bats as the primary reservoir [1,2], the ancestral origin of MERS-CoV has not been ascertained yet despite its close relationship with some bat coronaviruses [3,4,5]. Molecular assays play a crucial role in the diagnosis of emerging viruses such as Zika virus, which may overcome the limitations of viral cultures and serological tests, allowing more convenient, rapid, and accurate diagnosis. Immense research efforts have also been recently made to reveal the diverse virulence mechanisms and host–pathogen interactions of emerging viruses. On the other hand, with only a few exceptions such as influenza virus, treatment of emerging viruses is mostly supportive, as a result of the lack of effective antivirals for most of these viruses. Discovery of novel antiviral agents is eagerly awaited for many emerging viruses, though identification of antiviral targets which spare the host replication machinery can be difficult. Therefore, new research directions are crucial to predict, prevent, and combat virus diseases.

In this special issue, “Molecular Research on Emerging Viruses: Evolution, Diagnostics, Pathogenesis, and Therapeutics”, insights into advances and discoveries in understanding the different aspects of various emerging viruses are given by eight original studies and four review articles.

Three articles focus on arthropod-borne viruses (arboviruses) which are important emerging pathogens having caused various epidemics in recent years. In the systematic review and meta-analysis study by Coelho et al., the prevalence of microcephaly in infants born to Zika virus-infected women among all pregnancies was estimated [6], which may contribute to the understanding of the public health impact of this emerging arbovirus. The article by Le Coupanec et al., studies the viral replication during co-infection of Chikungunya and Dengue viruses which has been observed in some patients [7]. Co-infection with both viruses in *Aedes aegypti* mosquitoes was found to facilitate viral replication, suggesting the importance of pathogen–pathogen interactions. In another article, Lu et al. investigated the antiviral activity of histone deacetylase (HDAC) inhibitors as host-targeting agents against Japanese encephalitis virus (JEV) [8]. Tubacin, a selective HDAC6 inhibitor was found to be a potential host-targeting agent, demonstrating preventive and therapeutic activities against JEV infection. 

Zoonotic influenza viruses remain a significant concern to both human health and food industry, with their tendency to re-assort and mutate to generate novel strains capable of interspecies transmission. Yet, it remains difficult to predict the emergence potential of new strains. In the article by Eng et al., a machine learning approach was taken to build a zoonotic strain prediction model which could classify avian, human, or zoonotic strains with an estimated zoonotic risk [9]. In another article, Zhang et al. investigated the role of swine cellular microRNAs in regulating swine pandemic H1N1/2009 influenza A virus (SIV-H1N1/2009) replication [10]. Two microRNAs, ssc-miR-204 and ssc-miR-4431, were found to target viral haemagglutinin (HA) and non-structural protein (NS), respectively, and inhibit viral replication, providing insights into virus–host interaction and control of the virus in swine population.

Hepatitis viruses pose significant disease burdens worldwide and some have emerged or re-emerged in different populations. In the review article by Sridhar et al., the genotypic diversity and evolution of existing hepatitis E virus strains are reviewed, with a special focus on the emergence of camel hepatitis E variants [11]. In another article, Lee et al. reported novel hepatitis B virus intergenotypic recombinants from a patient co-infected with genotype A2 and C2 [12]. The results may prompt further studies on the clinical implications of such novel recombinant virus strains. 

Coronaviruses have continued to emerge or re-emerge in the last two decades to cause epidemics in humans and animals. In the review article by Lin et al., current knowledge on the molecular evolution, pathogenicity, and epidemiology of infectious bronchitis virus, which poses huge economic threats to poultry farms worldwide, was summarized [13]. 

Enteroviruses are another group of emerging viruses that can cause severe sporadic infections or epidemics especially in young children. In particular, enterovirus-D68 has emerged in recent years in various countries and is increasingly recognized as an important respiratory pathogen in the young and immunocompromised. In the article by Yip et al., the first fatal case of EV-D68 infection and genetic diversity of EV-D68 strains in Hong Kong were described [14]. The study also found a newly emerged subclade B3 and an interclade recombination between clade A and D2 strains in China. 

Two articles report on the virus–host interaction during porcine reproductive and respiratory syndrome virus (PRRSV) infection which causes severe losses in the swine industry worldwide. In one article, Ji et al. describes the role of porcine interferon stimulated gene 12a (*ISG12A*) in restricting PRRSV replication [15]. *ISG12A* was found to be upregulated in cells or tissues of pigs and could suppress PRRSV replication in infected MARC-145 cells, supporting its role in host immune response to PRRSV. In another article, Liang et al. describes the transcriptomic differences in porcine alveolar macrophages from two different pig breeds, Tongcheng and Large White pigs, in response to PRRSV infections [16]. Transcriptomics profiling of infected macrophages suggested that Tongcheng pigs, being more resistant to PRRSV infection, may promote the extravasation and migration of leukocytes from the capillaries to the surrounding tissues to defend against PRRSV and suppress apoptosis of macrophages in order to enhance antigen presentation. 

Last but not least, some mammalian arenaviruses are emerging viruses that may infect humans to cause lethal hemorrhagic fever. In the article by Ly, current knowledge on the differential immune responses to arenaviruses were summarized, which may help understand their pathogenesis and contribute to the development of vaccines [17].
